# The study on hemodynamic effect of series type LVAD on aortic blood flow pattern: a primary numerical study

**DOI:** 10.1186/s12938-016-0252-4

**Published:** 2016-12-28

**Authors:** Qi Zhang, Bin Gao, Yu Chang

**Affiliations:** School of Life Science and BioEngineering, Beijing University of Technology, Beijing, 100124 People’s Republic of China

**Keywords:** Series type LVAD, Swirling flow, Aorta, Blood flow pattern, Wall shear stress

## Abstract

**Background:**

Left ventricular assist device (LVAD) has become an alternative treatment for end-stage heart failure patients. Series type of LVAD, as a novel LVAD, has attracted more and more attention. The hemodynamic effects of series type LVAD on aortic blood pattern are considered as its important characteristics; however, the precise mechanism of it is still unclear.

**Methods:**

To clarify the hemodynamic effects of series type LVAD on aortic blood flow pattern, a comparative study on the aortic blood flow pattern and hemodynamic states were carried out numerically for two cases, including series type LVAD support and normal condition. The steady-state computational fluid dynamic (CFD) approach was employed. The blood flow streamline, blood velocity vector and distribution of wall shear stress (WSS) were calculated to evaluate the differences of hemodynamic effects between both conditions.

**Results:**

The results demonstrated that the aortic flow pattern under series type LVAD showed significant different from that of normal condition. The strength of aortic swirling flow was significantly enhanced by the series type LVAD support. Meanwhile, the rotating direction of swirling flow under LVAD support was also dominated by the rotating direction of series type LVAD. Moreover, the blood velocity and WSS under LVAD support were also significantly enhanced, compared with that under normal condition.

**Conclusion:**

The hemodynamic states, including the aortic swirling flow characteristic, was significantly dominated by LVAD support. Present investigation could provide not only a useful information on the vascular complications caused by LVAD support, but also provide a useful guide for optimal the structure of the series type LVAD.

## Background

Left ventricular assist devices (LVADs) have gradually become an alternative treatment for end-stage heart failure patient [1]. According to the connection configuration between the native heart and LVADs, two kinds of LVADs have been classified, named as bypass type LVAD and series type LVAD, respectively. Currently, the bypass type LVADs, which was bypassed with the native heart, has been widely used in the clinical practice. And their hemodynamic effects on the aorta have attracted more and more attentions. For instance, Karmonik et al. [2] firstly investigated the hemodynamic effects of LVADs cannula on the ascending aorta. It demonstrated that the wall shear stress (WSS) is significantly regulated by the position of cannula of LVADs. Subsequently, Karmonik et al. [3] compared the hemodynamic differences in ascending aorta between continuous and pulsatile flow LVADs. It was demonstrated that under pulsatile flow LVADs support, lower WSS and reduced pressure in ascending aorta was achieved. Caruso et al. [4] studied the effect of the height of the anastomosis on the aortic hemodynamic states. The results suggested that the placement of the outflow graft at 2 cm above the ST junction gave the most favorable hemodynamic profile.

**Fig. 1:**
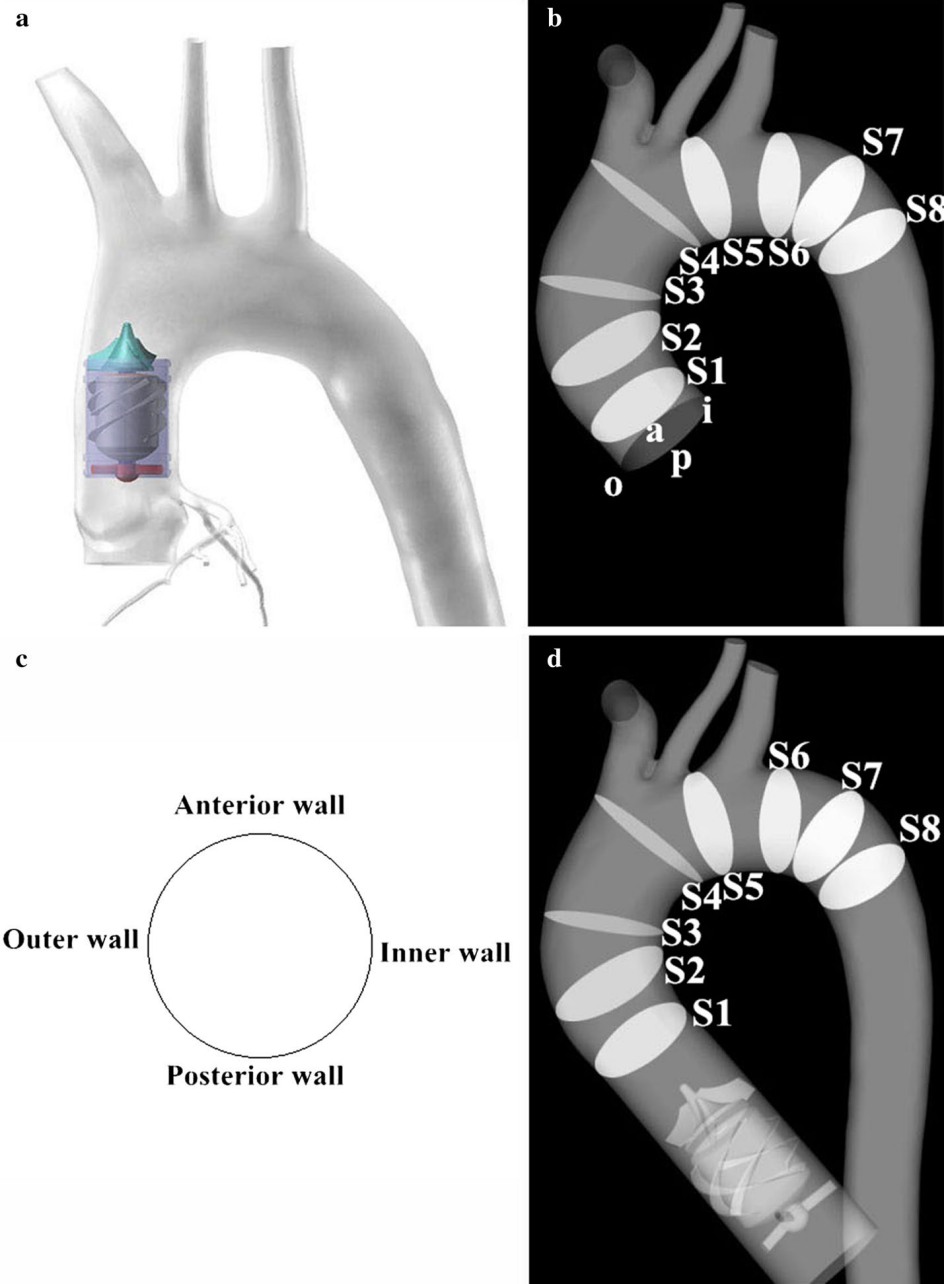
**a** The scheme of implantation position of series type LVAD. **b** The patient-specific aortic model. **c** View for flow pattern presentation. *a* anterior wall, *i* inner wall, *p* posterior wall, *o* outer wall. **d** The aortic geometric model with series type LVAD support

Along with the progress of LVAD, another novel kind of LVAD, implanted between the aortic root and aortic arch is gradually emerged (Fig. 1a) [5]. This kind of LVAD is named as series type LVAD. As the special implanted position, its hemodynamic effects on the aorta have attracted widely concerns. For instance, Gao et al. proposed the blood assist index (BAI) to evaluate the hemodynamic effect of series type LVAD on the left ventricle [6–9]. Moreover, the hemodynamic effect of series type LVAD on the cardiovascular system has been studied by using CFD approach and lumped parameter model method [10–14]. Although these studies focus on the hemodynamic effects of series type LVAD on cardiovascular system, the realistic LVAD outflow profiles are replaced by the ideal flow rate or pressure boundary condition in these studies. And the realistic blood flow pattern under series type LVAD support is still unclear.

Swirling flow has been confirmed as an important aortic hemodynamic factor, maintaining the normal aortic structure and function. Yashiro et al. [15] firstly reported that the swirling flow in the aorta had significantly effect on the remodeling of aortic tissue. Similarly, Stonebridge et al. [16] confirmed that aortic swirling blood flow dynamics is an emerging behavior that is common to normal individuals by using four-dimensional phase-contrast magnetic resonance imaging. In this study, the local normalized helicity (LNH) was proposed for evaluating the strength of aortic swirling flow. Subsequently, Deng et al. [17] reported that the swirling flow is desirable to make the blood flow stability and reduce turbulence, and to make the vascular walls to get smooth scour, reduce harmful substances in the vessel wall deposits and significantly increase the wall shear stress in the aorta and reduce the area of aortic blood flow stagnation zone [18]. Stonebridge [19] reported that swirling flow could more efficiently transport blood. Although above-mentioned studies demonstrated that the swirling flow has significantly benefit for maintaining normal aortic structure and function, the precise effect of series type LVAD support on the aortic swirling flow characteristic was still unclear. As series type LVAD was implanted into the ascending aorta, it may have more effect on the hemodynamic states and aortic swirling flow. Hence, there is a hypothesis that the series type LVAD support could cause significantly changes in swirling flow characteristic and aortic hemodynamic states, which may be contribute to relative aortic complications after LVADs support.

In order to test this hypothesis, CFD simulations were conducted to clarify the changes in aortic hemodynamic states and swirling flow characteristic. A patient-specific aortic geometric model with series type LVAD was reconstructed based on CT data of heart failure patient. Two static CFD simulations, including series type LVAD support case (named as LVAD case) and normal physiological condition (named as normal case), were conducted. The blood flow pattern, wall shear stress (WSS) and strength of swirling flow were calculated.

## Methods

The method, used in this investigation, is similar with that reported in the previous paper [20] of our team. And the details were described here for more clearly for the readers.

### Geometric model reconstruction

In order to obtain the change in hemodynamic states, two patient-specific aortic models with and without series type LVAD support were reconstructed based on a series of computed tomography angiography (CTA) images. The 3D patient-specific aortic model was reconstructed by using these images and by using commercial 3D reconstruction software MIMICS (Materialise, Belgium). And then, the model was send into software Geomagic (Geomagic, USA) to improve the surface quality of the aorta (Fig. 1b).

The series type LVAD was implanted into the ascending aorta [5]. It is a kind of axial flow pump, which consisting of anterior impeller, impeller and the rear impeller. The blood was pumped from the left ventricle to the aorta utilizing the rotation of impeller. The diameter of series type LVAD was 24 mm, which is consistent with the diameter of ascending aorta. Then the geometric model of series type LVAD, constructed by Solidworks 2014 (Dassault Systemes S.A, USA), was assembled with the aortic model by using commercial software FreeForm (Geomagic, USA) (Fig. 1d).

### Meshing generation

**Fig. 2:**
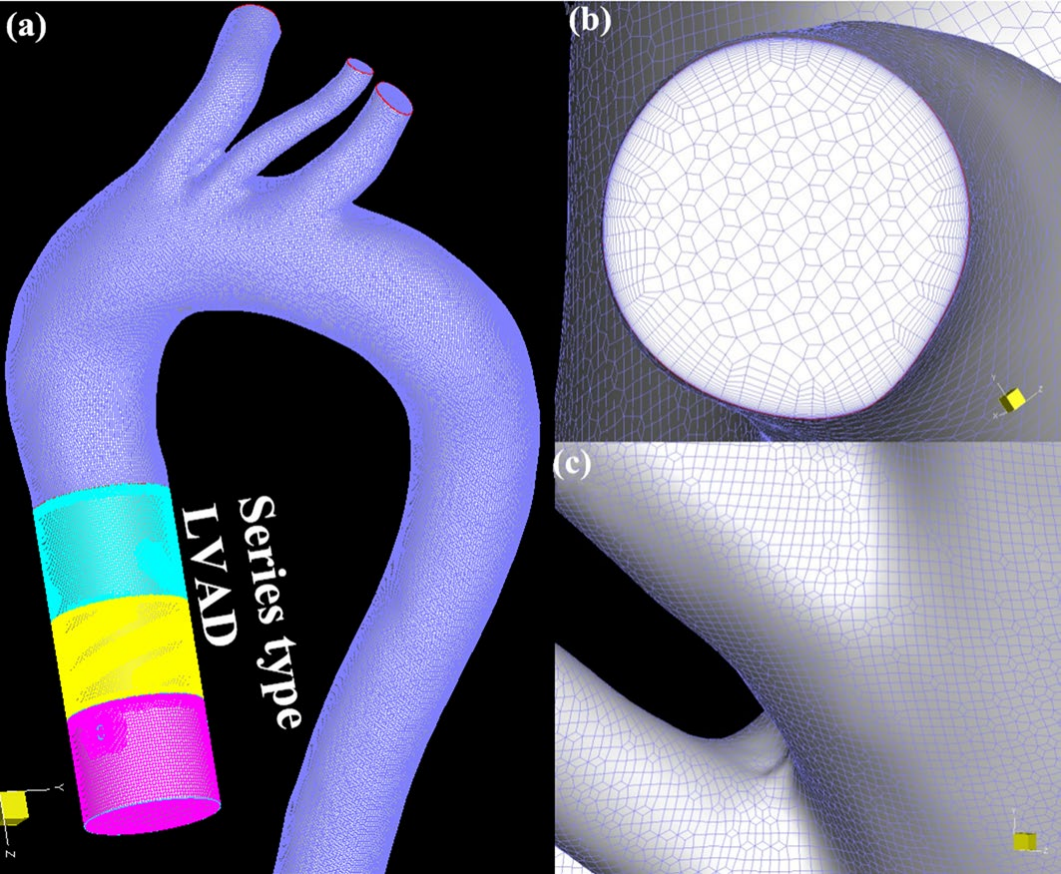
The finite element model used in this study

Both geometric models were meshed by using the grid generator Hexpress (Numeca, Belgium), which could provide high quality structural hexahedron grid for complex geometric model, such as the impeller and the aorta. In order to determine the appropriate numbers of grids for this model, grid independency tests, which target the flow rate in the descending aorta, are conducted. The results demonstrated that 10 million hexahedral elements (aorta 3.2 million elements, series type LVAD 7 million elements) are sufficient for this study (Fig. 2).

### Numerical approaches

In this study, the blood is assumed to be the incompressible, homogeneous and Newtonian fluid. The flow simulation was based on the momentum and mass conservation for incompressible fluid, known as the Navier–Stokes Eqs. [21] (1) and (2).

1 $$\nabla \cdot {\mathbf{u}} = 0$$

2 $$\rho \frac{{\partial {\mathbf{u}}}}{\partial t} + \rho ({\mathbf{u}} \cdot \nabla ){\mathbf{u}} = - \nabla p + \mu_{t} \nabla^{2} {\mathbf{u}}$$

where **u** is velocity vector, *t* represents the time, and *p* denotes the pressure, *ρ* and *μ*_*t*_ are the density and the turbulence viscosity of the blood. The Navier–Stokes equations were solved in NUMECA FINE/OPEN 5.1 (Numeca, Belgium), utilizing “Frozen Rotor” method, a second-order up-winding scheme and finite volume-based pressure-correction algorithm for the convective derivatives.

### Calculation settings

The blood is assumed to be the homogeneous, incompressible and Newtonian fluid, of which the density and viscosity is set 1050 kg/m^3^ [22] and 0.0035 Pa s [23], respectively. Aortic wall was assumed to be no-slip rigid. The simulation was carried out under static-state flow conditions, in which the inlet volume flow rate was set to be 5 L/min, and the zero-pressure boundary condition were assigned at all of outlets of the model. In addition, the rotational speed of LVAD was set to be 5000RPM, according to the inlet volume flow rate (5 L/min) and the rotating direction was counter clockwise. The convergence precision in this study is set to be 10^−3^.

### k-w SST turbulence model

Based on the geometric size of aorta, the blood density and the blood flow velocity, the peak Reynolds number Re is larger than 5000. Hence, the k-w SST turbulence model, proven to be fit for illustrating the turbulence flow states in aorta and series type LVAD [24], was used in this study. The inlet turbulence intensity of the aorta is 1.5% in this study. The k-w SST turbulence model denoted as Eqs. (3), (4) and (5) [25]. This model assumes that the turbulence viscosity is linked to the turbulence kinetic energy and the turbulent frequency or specific dissipation rate *ω*, through

3 $$\mu_{t} = \rho \frac{k}{\omega }$$

This model solves two transport equations, one for *k* and another for *ω*. The *k* equation is

4 $$\rho \frac{\partial k}{\partial t} + \rho U_{j} \frac{\partial k}{{\partial x_{j} }} = \tau_{ij} \frac{{\partial U_{i} }}{{\partial x_{j} }} - \beta^{*} \rho k\omega + \frac{\partial }{{\partial x_{j} }}\left[ {(\mu + \sigma^{*} \mu_{t} )\frac{\partial k}{{\partial x_{j} }}} \right]$$

while the specific dissipation rate equation *ω*, is as such

5 $$\rho \frac{\partial \omega }{\partial t} + \rho U_{j} \frac{\partial \omega }{{\partial x_{j} }} = \alpha \frac{\omega }{k}\tau_{ij} \frac{{\partial U_{i} }}{{\partial x_{j} }} - \beta \rho \omega^{2} + \frac{\partial }{{\partial x_{j} }}\left[ {(\mu + \sigma_{\omega } \mu_{t} )\frac{\partial \omega }{{\partial x_{j} }}} \right] + 2(1 - F_{1} )\rho \sigma_{\omega } \frac{\partial k\partial \omega }{{\omega \partial x_{j} \partial x_{j} }}$$

## Results

In order to evaluate the hemodynamic effects of series type LVAD support on the aortic hemodynamics, a comparison between LVAD case and normal case were conducted. The streamline, contour of blood velocity, blood velocity vector, area average helical density (Ha) and WSS were discussed. In addition, the maximum value, minimum value and mean value of blood velocity, WSS and Ha were list in the Table 1.

**Table 1:** The results of the numerical simulation

	Velocity (m/s)			WSS (Pa)			Hd (m/s^2^)		
	Max	Min	Mean	Max	Min	Mean	Max	Min	Mean
LVAD case	1.73	0	0.23	30	0	4.89	793	−2220	3.65
Normal case	0.58	0	0.14	8	0	2.046	90	−67	0.15

*WSS* wall shear stress, *Hd* helical density

### Streamline of blood flow

**Fig. 3:**
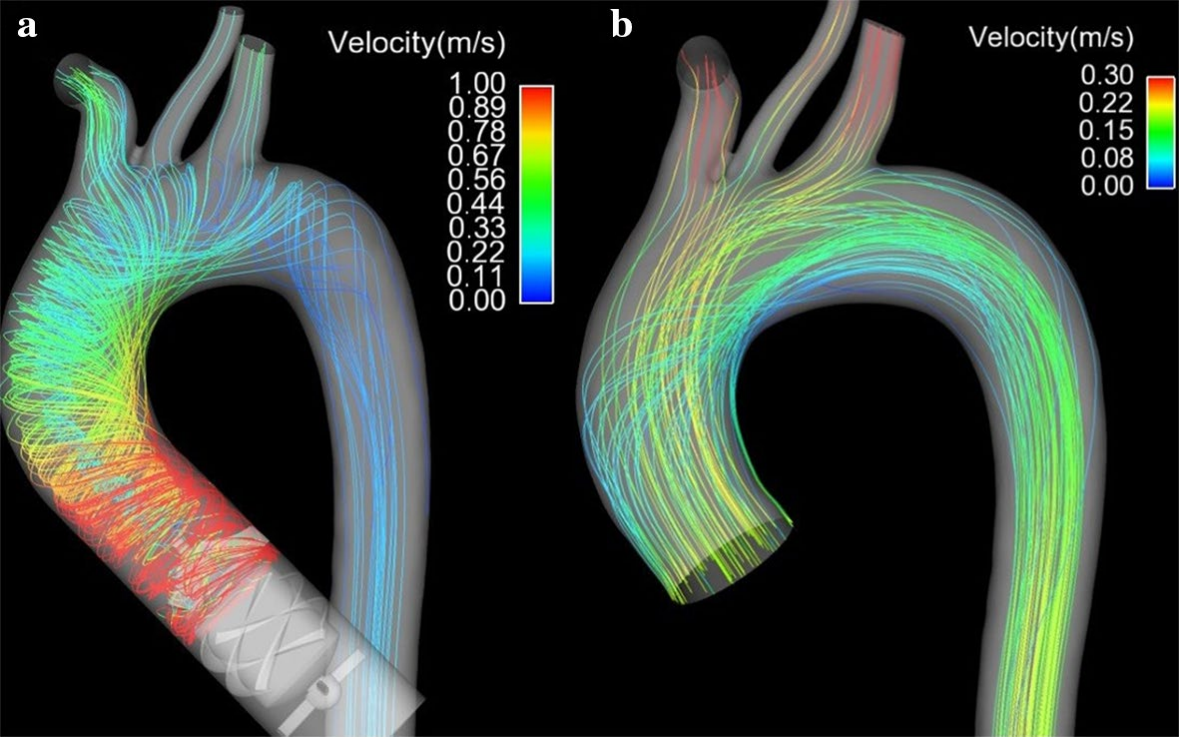
The streamline of blood flow in the aorta under series type LVAD support and normal condition. **a** Illustrate the obvious swirling flow in the ascending aorta under series type LVAD support. **b** Shows the normal blood flow pattern in the aorta under normal condition

Figure 3 illustrated the streamline of blood flow under both LVAD case and normal case. Figure 3a is the streamline of blood flow under LVAD case. And Fig. 3b is the streamline of blood flow under normal case. From Fig. 3, it is found that the magnitude of blood flow under LVAD case was significantly higher than that of blood flow under normal case (LVAD case 1.0 m/s vs. normal case 0.3 m/s). Besides of that, the flow pattern in the aorta is also significant different between LVAD case and normal case. Under LVAD case, the obvious swirling flow, whose direction is counter clockwise, was observed in the ascending aorta and aortic arch. The direction of swirling flow is consisting with the rotational direction of series type LVAD. In contrast, under normal case, the obvious swirling flow phenomenon is observed at the aortic arch, rather than ascending aorta. Meanwhile, the direction of swirling flow is clockwise.

### Velocity profiles of blood flow

To facilitate the illustration of the velocity field under cases, the velocity contour and vector on the varied sections were discussed.

**Fig. 4:**
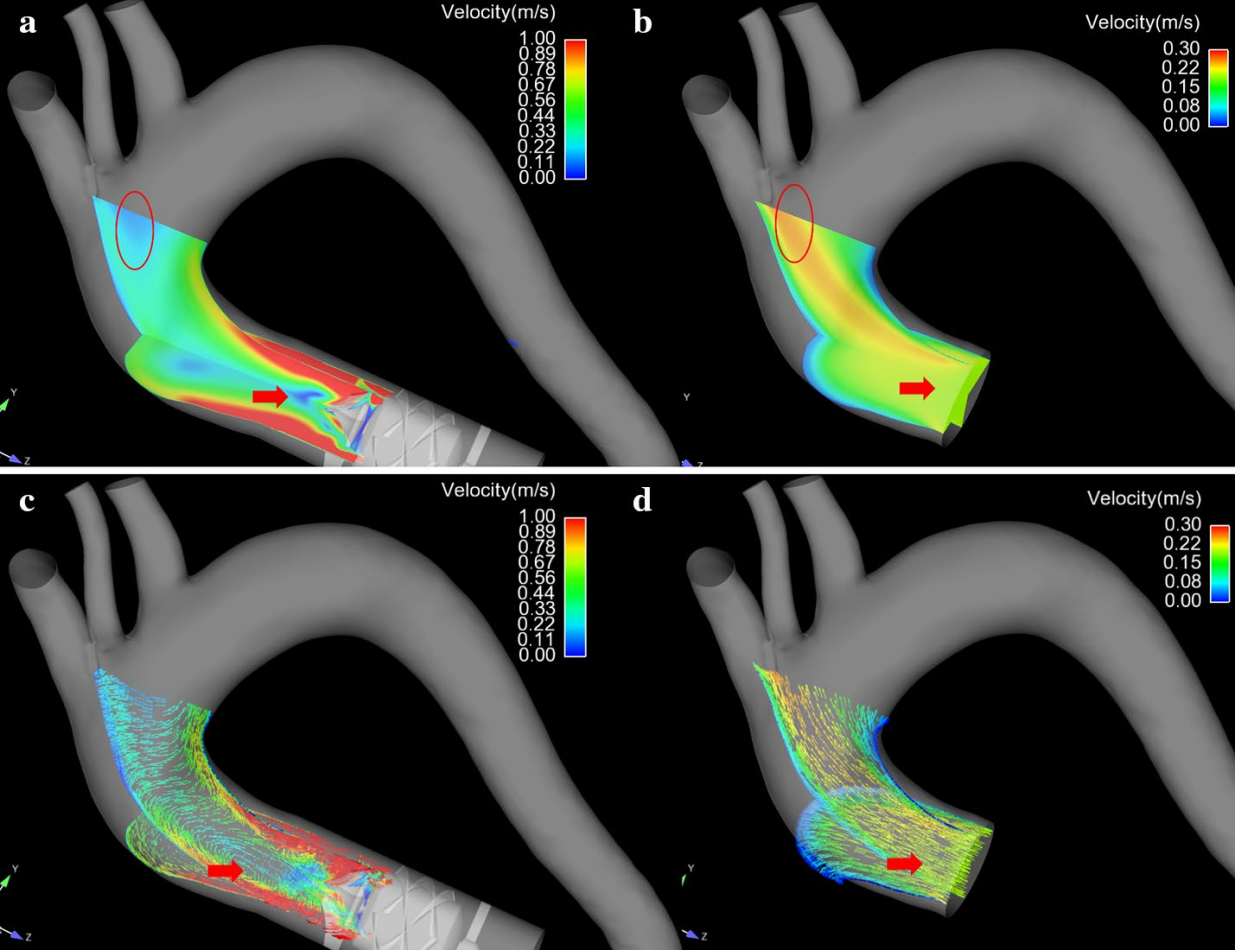
The velocity contour curve and vector curve of blood flow in the ascending aorta under both case. **a** Illustrate the blood velocity contour plane under series type LVAD support. **b** The blood velocity contour plane under normal condition. **c** Shows the blood velocity vector in the ascending aorta under series type LVAD support. **d** The blood velocity vector under normal case

Figure 4 illustrated the blood velocity contour and vector distribution at the ascending aorta under both cases. Figure 4a shows the velocity contour distribution of blood flow at the ascending aorta under LVAD case. Figure 4b is the blood velocity contour distribution at ascending aorta under normal case. Figure 4c illustrates the blood velocity vector at the ascending aorta under LVAD case. Figure 4d shows the blood velocity vector at ascending aorta under normal case. From Fig. 4, it is seen that the blood flow pattern under LVAD case is quite different from that under normal case. Under LVAD case, an obvious low velocity region was observed at the ascending aorta (Fig. 4a, red arrow, 0.058 m/s), while there is not similar blood velocity distribution under normal case (Fig. 4b red arrow, 0.2 m/s). Moreover, an obvious vortex and stagnant region were observed at the ascending aorta under LVAD case (Fig. 4c red arrow), compared with that under normal case (Fig. 4d, red arrow). Besides that, the high blood velocity location under LVAD case is also quite different from that under normal case. Under normal case, the high blood velocity region is at the center of the aortic lumen (Fig. 4b red circle, 0.26 m/s), which is consisting with the hemodynamic theory. However, under LVAD case, the high blood velocity region is at inner wall of the aorta (Fig. 4a, red circle, 0.16 m/s).

**Fig. 5:**
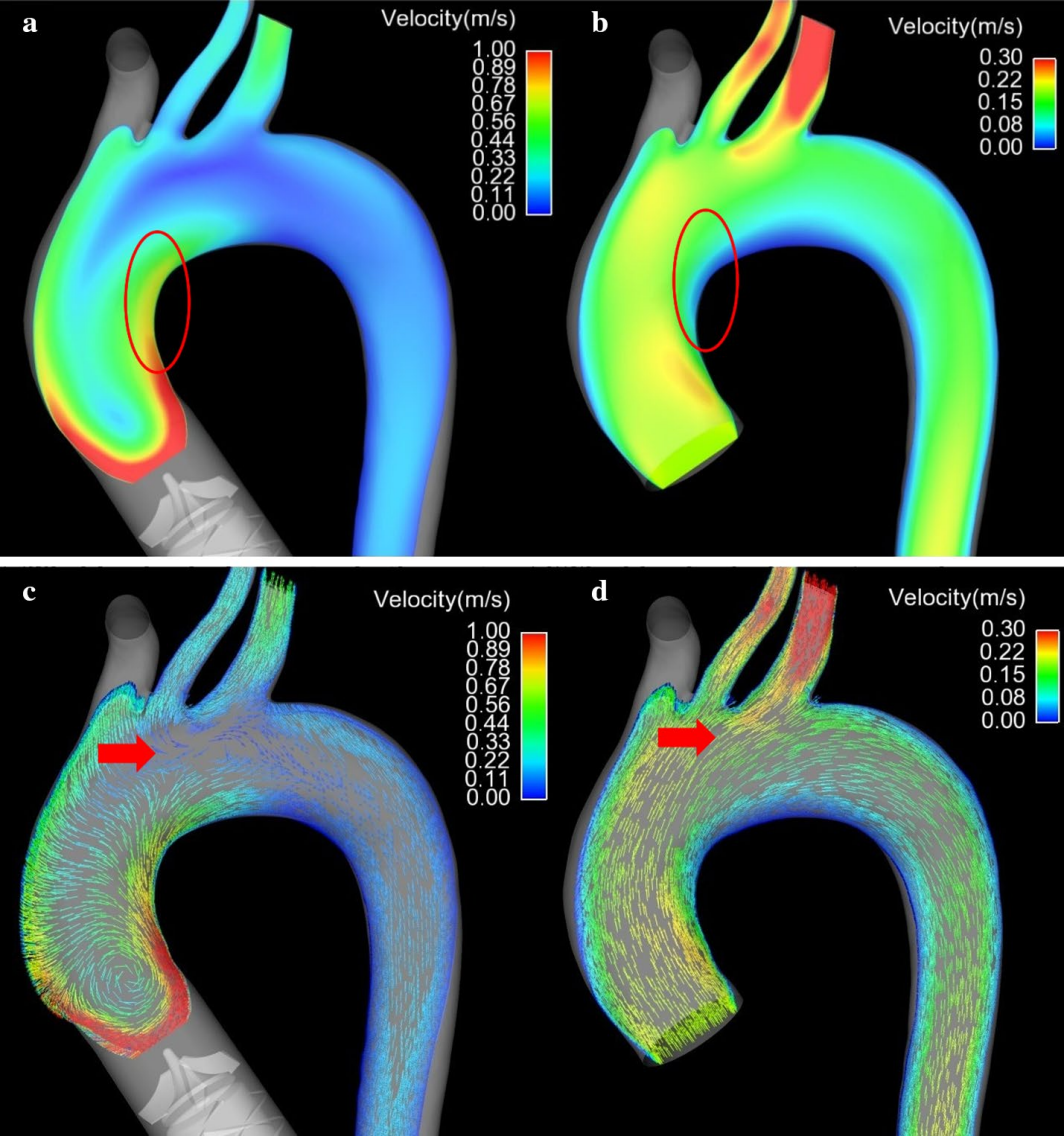
The blood flow pattern in the aorta arch. **a** The blood velocity contour plane under series type LVAD support. **b** The blood velocity contour plane under normal case. **c** The blood velocity vector in the aorta under series type LVAD support. **d** The blood velocity vector under normal case

Figure 5 illustrates the blood velocity distribution at the aortic arch. Figure 5a is the blood velocity contour under LVAD case. Figure 5b is the blood velocity contour under normal case. Figure 5c shows the blood velocity vector under LVAD case. Figure 5d illustrates the blood velocity vector under normal case. Under normal case, an obvious low velocity region was seen at the inner wall of the aorta (Fig. 5b, red circle, 0.05 m/s), while under LVAD case, the blood velocity at the same region is significant higher than that under normal case (Fig. 5a, red circle, 0.76 m/s). Besides that, an obvious vortex was observed near the base of left subclavian artery (Fig. 5c, red arrow), while there is no similar flow pattern under normal case (Fig. 5d, red arrow).

### Swirling flow profiles

**Fig. 6:**
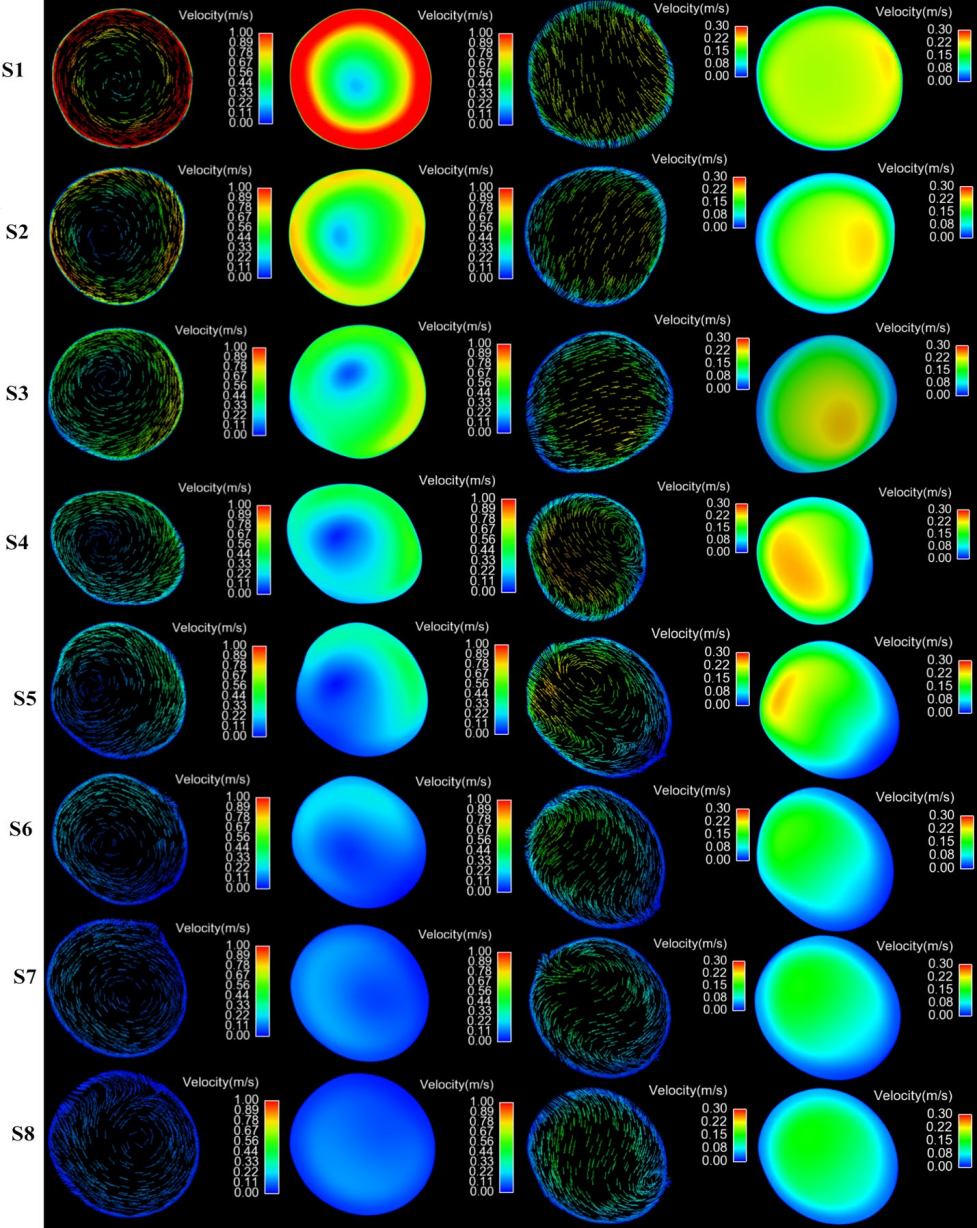
The contour and vector of blood velocity at cross sections (*S1*–*S8*) under both cases. The *left hand columns* show the blood vector and contour under LVAD case. The *right hand columns* show the blood vector and contour under normal case

To facilitate the presentation of the swirling flow profiles in the aorta, eight representative slices were selected along the longitudinal direction of the centerlines shown in Fig. 1. Slice 1 (S1) is located at the front part of ascending aorta. Slice 2 (S2) is located at middle way of the ascending aorta. Slice 3 (S3) is at behind part of ascending aorta. Slice 4 (S4) is locate at the entrance of aortic arch. Slice 5 (S5) is between the left common carotid artery and the left subclavian artery. Slice 6 (S6) is at the exit of the aortic arch. Slice 7 (S7) is at the entrance of descending aorta. Slice 8 (S8) is at the anterior part of descending aorta. The view of the velocity contour and vector for all slices is show at Fig. 6.

Under normal case, the blood flow at the inlet of the model is plat. However, as the blood move towards the S1, the blood flow skew towards the inner wall of the aorta (Fig. 6 S1 normal case) and the high velocity region is near the inner wall. While under LVAD case, an obvious swirling flow, which direction is consisting with the rotational direction of LVAD, is observed at S1 plane. And the high blood velocity region distribute uniformly near the aortic wall (Fig. 6 S1 LVAD case). When the blood flow reached to the entrance of the aortic arch (S4), there was a vortex between the anterior wall and inner wall, and the high velocity region is towards between the outer wall and posterior wall forming a crescent shape (Fig. 6 S4 normal case). In contrast, under LVAD case, the rotating flow, whose direction is counter clockwise, is still obvious, and the high velocity region is towards between the anterior wall and inner wall (Fig. 6 S4 LVAD case). With the blood flow moving farther down, more complex flow pattern was observed at meddle way of the aortic arch (S5). Under normal case, two vortexes are seen at S5. The one is near the anterior wall and the other one is near the outer wall (Fig. 6 S5 normal case). While under LVAD case, a uniform rotating flow was seen at S5, and the core of the flow was toward the outer wall. Moreover, under normal case, along with the blood flow enter into the descending aorta (S6, S7, S8), the strength of the swirling flow, form in the aortic arch, attenuated and the blood velocity gradually become uniform.

**Fig. 7:**
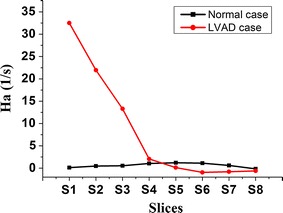
The change in Ha along with the longitudinal direction of the centerlines of aorta

In order to further study the difference in aortic swirling flow characteristics, the change in area average helical density (Ha) between normal case and LVAD case was shown in Fig. 7. The result demonstrated that LVAD support significantly enhanced the aortic helical density of blood in the ascending aorta and aortic arch; while the aortic helical density of blood was weaken in the descending aorta. In addition, under LVAD case, Ha is gradually decreased along with the longitudinal direction of the centerlines of the aorta. In contrast, Ha gradually increases in the ascending aorta, and reaches its maximum at aortic arch, while gradually decreases in the descending aorta.

### Distribution of WSS

**Fig. 8:**
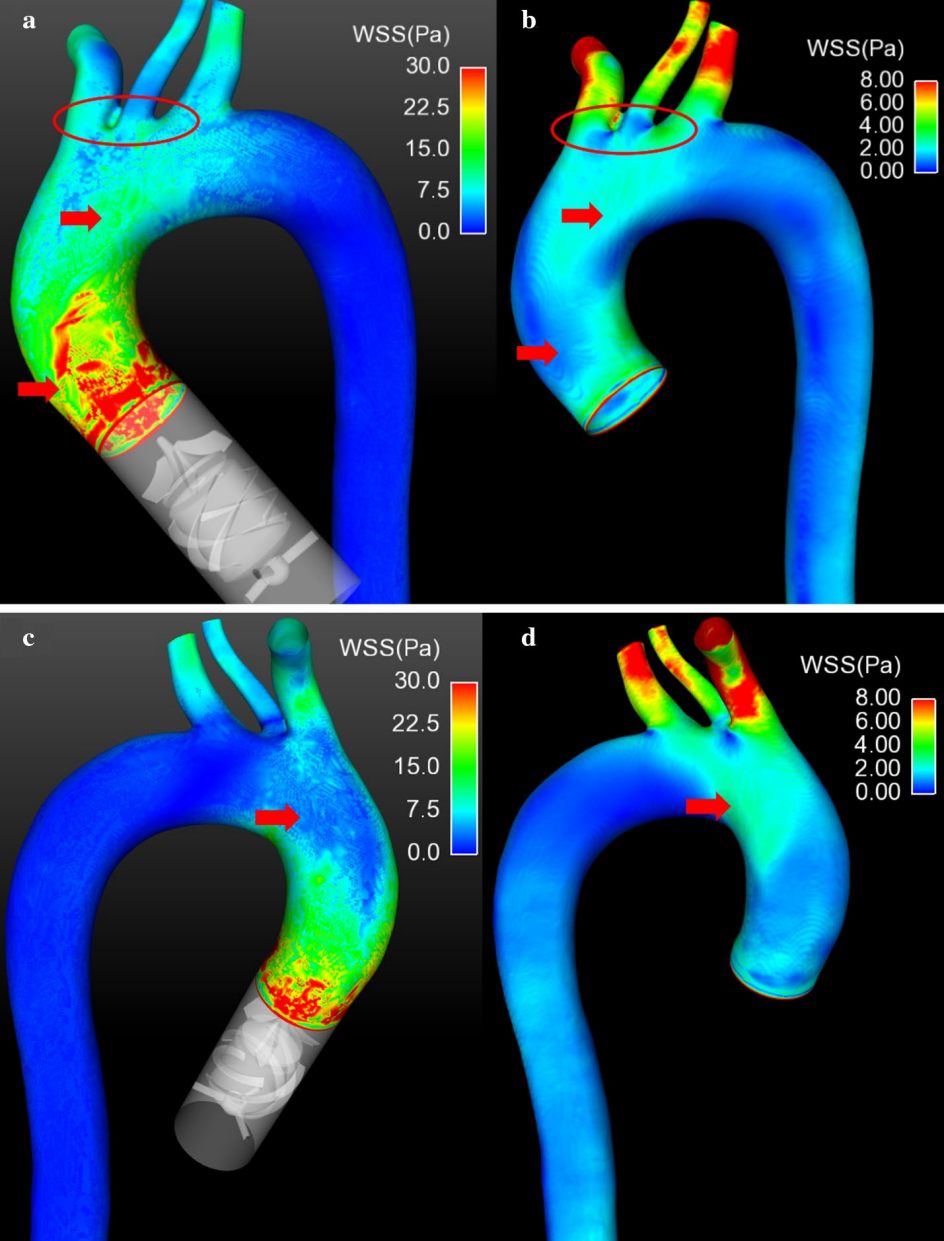
The contour of WSS under both cases. **a**, **c** Illustrate the WSS distribution under series type LVAD support. **b**, **d** Show the WSS distribution under normal case

To illustrate the distribution of WSS, the contour of WSS under both cases was presented in Fig. 8. Figure 8a, c is the distribution of WSS under LVAD case. Figure 8b, d is the distribution of WSS under normal case. It is seen that there are some difference between both cases. Overall, under LVAD case, the magnitude of WSS is significantly higher than that under normal case (LVAD case 30 Pa vs. normal case 8 Pa). And the highest WSS region, under LVAD case, focus at the ascending aorta (Fig. 8a, b red arrow). In addition there is a low WSS region at the base of brachiocephalic artery and left common carotid artery under normal case (Fig. 8b, red circle, 0.46 Pa), while the same region is high WSS under LVAD case (Fig. 8a red circle, 12.3 Pa). Moreover, there is a relative low WSS region at the posterior wall near the entrance of aortic arch (Fig. 8c red arrow, 7.0 Pa), while under normal case, the WSS distribution at the same region is relative high (Fig. 8d red arrow, 4.3 Pa).

## Discussion

Swirling flow has been considered as an important physiological flow phenomenon in the aorta. Many studies on the physiological role of swirling flow have been conducted. The results demonstrated that swirling flow have benefit for achieving uniformity of wall shear stress [26], inhibiting flow stagnation [27] and reducing the surface concentration of low-density lipoprotein [18] and increasing the flux of oxygen [28]. Although there are many studies on the swirling flow in the aorta, to our knowledge, there has been no study that has analyzed the effect of LVAD support on the aortic swirling flow characteristic. The present numerical study has demonstrated that the series type LVAD could significantly change the aortic blood flow pattern, the swirling flow characteristic and WSS distribution. The strength of swirling flow, under LVAD case, is significantly higher than that of swirling flow under normal case. In addition, the high velocity blood flow, under LVAD case, was observed near the inner wall, while, under normal case, high velocity blood flow was observed at the center region of aortic lumen.

The swirling blood flow in the human aortic lumen is believed to be a typical example of “form follows function”, and it is hypothesized that swirling flow could provide guaranties for the inner surface of the ascending aortic wall to get smooth and even a flushing by the blood so that the atherosclerotic plaques can hardly form in the area of ascending aorta [18]. Hence, the swirling flow could provide much benefit for maintaining aortic function. And along with the decrease of the strength of swirling flow, the areas of low blood velocity region become bigger [29] and the level of polarized low density lipoprotein in the aorta is worse [30]. Similarly, the swirling flow was confirmed to reduce the luminal surface low density lipoprotein (LDL) concentration in the aortic arch [18] and probably played a role in suppressing severe atherosclerosis [31] and in regulating vascular smooth muscle cell function [32]. The present study demonstrated that the strength of swirling flow is significantly enhanced after LVAD support (Fig. 6), which means the series type LVAD may promote the benefit of swirling flow. These changes maybe improve the complications caused by conventional LVAD support, such as peripheral vascular dysfunction [33] and impaired vascular compliance [34]. In addition, from Fig. 3a, it is seen that the rotating direction of blood flow under LVAD case is counter clockwise, which is consistent with the rotating direction of LVAD. However, the swirling flow generated by aortic geometrics is mainly clockwise [31]. And according to Chien’s study, the flow pattern could determine the arrangement and phenotype of endotheliocyte [35]. Hence the change in the rotating direction of swirling flow in the aorta may lead to the remodeling of aorta. And the precise effect of it will be study in the future.

Meanwhile, the high blood velocity region, under LVAD case, is near the aortic wall (Fig. 4a), rather than that in the center of aortic lumen, under normal case (Fig. 4b). That means, under LVAD case, the flush effect is significantly enhanced so that the atherosclerotic plaque and low-density lipoprotein will more difficult to deposit on the aortic surface compared with that under normal case. Moreover, the results demonstrated that under LVAD case the WSS at the ascending aorta is significant higher than that under normal case. Meanwhile, WSS is an important role for regulating the arrangement and function of endotheliocyte [35]. Zhang et al. report that the magnitude [22] and frequency [23] of WSS could significantly change the level of inflammation and oxidative stress of endotheliocyte. Similarly, Chakraborty et al. [36] demonstrated that the directionality of WSS is an important determinant of cellular responses, which could regulate the proliferation, morphology and genetic expression of endothelial cells. In this study, the WSS distribution, under LVAD case, is quite different from that under normal case. Under LVAD case, the highest WSS regions mainly locate at the ascending aorta, while the distribution of WSS is even (Fig. 7a, b, red circle). This may due to the velocity of blood flow, jetting out from series type LVAD, is significantly higher than that jet from the left ventricle. And the blood velocity at the aortic wall is considered as zero. Hence the velocity gradient, under LVAD case, is higher than that under normal case. Dolan et al. [37] reported that the excessive WSS show to trigger aneurysm initiation of endothelial cells. Hence, for series type LVAD, the blood velocity gradient at the outlet of LVAD should be paid more attention, when the diffusor was designed. In addition, under LVAD case, WSS at the base of brachiocephalic arterial and left common carotid artery are more uniform than that under normal case, which may reduce the incidence of endothelial dysfunction [38]. Note that under LVAD case, the WSS near the posterior wall is lower than other positions. That may result from the interaction between counter clockwise swirling flow and the complex aortic geometry. Hence, this phenomenon provide an important information to the designer of series type LVAD that the strength and direction of swirling flow should be considered as an important optimal target during the series type design.

## Limitation

In this study, the steady-state CFD simulation was conducted to evaluate the hemodynamic differences of aortic hemodynamic states between LVAD support and normal condition. Although the transient boundary condition was confirmed to provide more accuracy hemodynamic state, the steady-state CFD simulations were also widely applied. This study focuses on the hemodynamic differences of aorta between series type LVAD support and normal condition. Hence, the steady-state CFD simulation could provide sufficient hemodynamic information for researcher. And in the future, the unsteady-state CFD simulation will be conducted to clarify the realistic aortic hemodynamic states under series type LVAD support.

In this study, the zero-pressure is chosen as the outlet boundary condition. This is due to the deformation of aortic wall was neglected, and the blood flow pattern in the aorta mainly determined by the pressure gradient. Hence, the zero-pressure boundary condition has little effect on the accuracy of the results.

In this study, the deformation of aortic wall was neglected for reducing computational cost. According to literatures, deformation of aortic wall pattern has little effect on the blood flow pattern. And in the future, the fluid–structure-interaction approach will be used to study the hemodynamic effect of series type LVAD on the aortic wall.

## Conclusion

In order to evaluate the hemodynamic effect of series type LVAD on the aortic flow pattern, a steady-state numerical study was conducted. The results demonstrated that series type LVAD could significantly change the hemodynamic states, especially the swirling flow characteristic, in the aorta. In addition, the rotating direction of swirling flow, in the aorta, is governed by the rotating direction of series type LVAD. The flush effect of blood flow at the ascending aorta is also significantly enhanced by series type LVAD support. Meanwhile, the WSS distribution is also changed by the series type LVAD support.

## Abbreviations

LVAD: left ventricular assist device
CFD: computational fluid dynamic
WSS: wall shear stress
LNH: local normalized helicity
CT: computed tomography
CTA: computed tomography angiography
Ha: area average helical density
